# A Curriculum Integrating STEAM and Maker Education Promotes Pupils' Learning Motivation, Self-Efficacy, and Interdisciplinary Knowledge Acquisition

**DOI:** 10.3389/fpsyg.2021.725525

**Published:** 2021-09-08

**Authors:** Yangyang Jia, Bing Zhou, Xudong Zheng

**Affiliations:** School of Educational Information Technology, Faculty of Artificial Intelligence in Education, Central China Normal University, Wuhan, China

**Keywords:** engineering design, STEAM education, Maker education, STEAM and maker integrated curriculum, learning motivation, self-efficacy, interdisciplinary knowledge

## Abstract

Contemporary society expects learners to synthesize large amounts of available information and take advantage of interdisciplinary knowledge to tackle complex, real-world issues. STEAM education aims to cultivate students' ability to solve such problems through interdisciplinary thinking but is often represented by courses that are merely disjointed arrays of school subjects. On the other hand, Maker education harnesses society's enthusiasm for technological innovation and creativity but overlooks the scientific principles that underpin these processes. This research presents a novel elementary school course informed by the interdisciplinary principles of STEAM, integrated with Maker's focus on technology and creativity. The course design also utilized engineering design as a meta-thematic framework. A total of 164 third-grade pupils participated in the research, with responses analyzed using descriptive statistical methods. The findings indicated that the integrated design of the course promoted pupils' learning motivation, self-efficacy, and acquisition of interdisciplinary knowledge. These effects were not gender-specific and demonstrate the potential applicability of a STEAM/Maker integrated approach to curriculum design in other settings.

## Introduction

As the information age gives way to the comprehensive age (Cai, 2011), learners are increasingly required to synthesize large amounts of information and employ interdisciplinary knowledge to solve complex real-world problems (Nadelson and Seifert, 2017). Complex Problem Solving (CPS) is deemed to be a key cross-curricular skill of the 21st century (Herde et al., 2016). However, much formal education has traditionally been premised on the division of knowledge into discrete subject areas. Although the division of knowledge into disciplines is conducive to scientific research (Morrison et al., 2009), it detaches formal education from the real world, meaning learners may fail to apply the knowledge they have learned to resolve practical issues. This, in turn, leads to the emergence of the phenomenon of “useless knowledge” (Linn and Hsi, 2000).

STEM education, guided by interdisciplinary thinking, has received extensive attention due to its focus on cultivating students' ability to solve complex and realistic problems (National Academy of Engineering and National Research Council, 2014). Scholars have increasingly realized that arts and humanities subjects help students understand the connections between different disciplines from a more comprehensive perspective (Watson and Watson, 2013; Kant et al., 2018), and STEM education has evolved into a new “STEAM Age.” While acknowledging the distinction between STEM and STEAM, this is not a central concern of the present study.

In essence, STEM education entails an interdisciplinary approach oriented toward science and engineering education, guided by the concept of knowledge integration. However, in practice, it often results in “patchwork” curricula stitched together from several different subjects, which runs counter to its stated aim of achieving greater disciplinary integration (Thuneberg et al., 2017). STEM education eradicates the barriers between themes and prioritizes current tools and technical design to resolve complex contextual problems (Kennedy and Odell, 2014). Formerly, science and mathematics were approached as isolated subjects (Breiner et al., 2012; Quigley and Herro, 2016), with almost no consideration of technology or engineering (Hoachlander and Yanofsky, 2011; Timms et al., 2018). Indeed, an atomized curriculum structure and the insufficiency of teachers' skills are the two critical reasons for STEM education's lack of success in practice, explaining its repeated and ongoing failure to achieve its intended goals (Blackley and Howell, 2015). Moreover, many curricula are not designed or delivered in ways that improve students' capacity to innovate (Taylor, 2016). School STEM programs are frequently characterized by fragmented courses whose focus is narrow (Kim and Park, 2012; Park, 2012) and whose effectiveness has not been adequately verified (Wang et al., 2018).

Maker education is a new type of educational practice which aims to foster creativity. It views learning as a shared, social process based on the design and production of physical objects (Halverson and Sheridan, 2014). It assumes that the joy of creation can stimulate students' curiosity (Anderson, 2012). Maker education focuses on the use of technical tools and equipment but is less concerned with developing knowledge of scientific concepts and principles (Dougherty, 2012).

Research indicates that STEAM education with Maker is potentially well-suited to classroom learning in the era of the Fourth Industrial Revolution (Kim and Kim, 2018). This raises the question of how to overcome the issues of disparate multidisciplinarity in STEM education and the neglect of scientific principles in Maker education to integrate the strengths of both approaches into classroom teaching. Maker education prioritizes design above processing (Jacobs and Buechley, 2013; Halverson and Sheridan, 2014) and includes the application of digital technology (Martin, 2015). These digital tools have greatly reduced experimental errors (Snyder et al., 2014), while at the same time improving the efficiency of hands-on practice (Lipson and Kurman, 2013), enabling student learning to proceed via a varied process of trial and error. However, the potential of Maker education is impacted by the current lack of genuinely interdisciplinary, unified approaches to teaching. As a result, learners' skills in and knowledge of the use of technical tools and equipment remain shallow and unintegrated. This contributes to an excessive emphasis on the value of manufactured products in what Chachra (2015) refers to as a deformed technological culture.

A complete engineering design is an emergent and highly iterative process that can facilitate meaningful learning (Roehrig et al., 2012; English, 2016). It provides a framework enabling the establishment of links between the various disciplines of STEM education (Fan and Yu, 2017) which can then be more closely integrated (Kelley and Knowles, 2016). It is well-suited to Maker's focus on the creative use of technology. Moreover, engineering-oriented STEM courses are best placed to instill the key concepts of STEM education and promote students' acquisition of content (Christensen and Knezek, 2017). However, the key task that remains is to develop syllabi that integrate STEM and Maker into classroom practice. The following account of an interdisciplinary STEM- and Maker- integrated curriculum in the field of engineering design addresses this task.

Engineering design is a creative, knowledge-driven process, in which the concepts of devices, systems or processes are generated, specified, and evaluated (Dym, 1994). During this process, specific constraints are balanced with the achievement of customers' goals and requirements (Dym et al., 2005). Engineering design includes but is not limited to the processes of questioning, imagination, creation, testing, and improvement (Dieter and Schmidt, 2009; Shahali et al., 2016). Its realization requires the use of scientific and mathematical concepts (Moore and Smith, 2014), so it can be used as the basis for establishing such concepts and practical connections in STEM education (Sanders, 2008; Donna, 2012). This also aligns it with the goal of disciplinary integration in K-12 STEM education (Moore et al., 2014). The considerable utility of engineering design as a meta-thematic concept (Fan and Yu, 2017) helps explain its considerable influence on STEM education (Katehi et al., 2009). Finally, engineering design is regarded as an essential ability for STEM students (Atman et al., 2007).

Moreover, engineering design overlaps with Maker's focus on transformative innovation in the field of technology. Maker education emphasizes the use of software and hardware to transform creativity into entities (Halverson and Sheridan, 2014). It enables students to transform the potential of their subjective initiative into real subjective creativity. At the same time, they can apprehend the potential power of scientific rationality to remold nature into concrete material power. Maker's interest in fostering technological innovation can be focused on specific learning projects by utilizing the concepts of engineering design. As a bridge between STEM and Maker, engineering design provides students with an opportunity to work on technological innovation while transforming abstract science and mathematics concepts into concrete practical processes, establishing links to real life, and improving students' familiarity with and interest in the disciplinary content (Clapp and Jimenez, 2016).

Interest is a prerequisite for students to participate in STEAM learning (Maltese and Tai, 2011; Maltese et al., 2014). And interest is closely related to intrinsic motivation, when individuals are intrinsically motivated, they do activities out of interest in the activity (Wigfield et al., 2012). Therefore, testing students' learning motivation is an important indicator of curriculum quality. Self-efficacy is an element of intrinsic motivation (Deci et al., 1981), which defined as judgment or assessment of one's capabilities to perform a particular given task successfully (Bandura et al., 1999). Self-efficacy is regarded as a major trigger for purposeful behavior and the perseverance to achieve set goals (Özcan and Eren Gümüş, 2019), which has been highlighted as an essential predictor of general academic performance (Ferla et al., 2009). For the above reasons, while testing interdisciplinary knowledge acquisition, this research will focus on the students' learning motivation and self-efficacy to reflect learning quality.

We are currently developing a series of curriculum with the integration of STEAM and Maker, aimed at the comprehensive training of students' knowledge, abilities, and literacy in K-12 stage. This paper reports the results of our first round of development, which including the following questions: (a) How can we design curriculum framework with the integration of STEAM and Maker based on the idea of engineering design? (b) How can we develop a curriculum based on the framework? (c) How to evaluate the effectiveness of the development curriculum?

## Framework For Developing an Steam and Maker Integrated Curriculum

**Figure 1:**
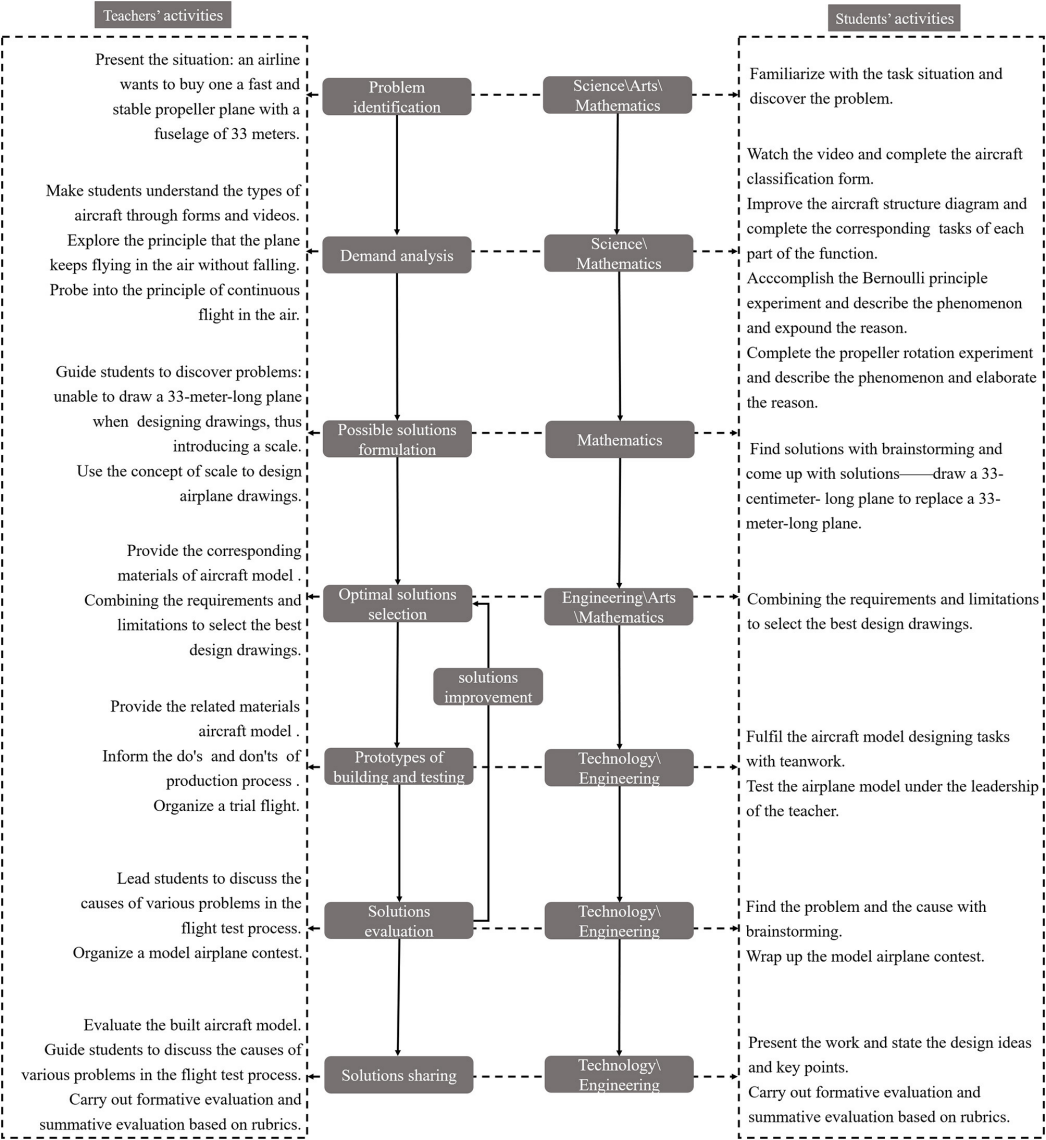
Framework for developing a STEAM and maker integrated curriculum.

The framework for the course content of *Soaring in the air* is shown in Figure 1. The syllabus is closely tied to the national curriculum standards for K-12 in China. The selection of themes draws on real-world scenarios and the content setting helps to ensure that students establish connections between disciplines. The purpose of the design activity is to allow students to use their brains in the hands-on process. The course's overarching aim is to allow students to turn the objects of their imaginations into real artifacts through practical, experiential learning. Key to this process is the students' ability to use their minds, rather than simple hands-on skills.

The curriculum design includes eight main steps of engineering design. First, clarify the problems to be solved in this course which is how to make a propeller aircraft with 33 m. Second, confirming the learning requirements. The reasons why an aircraft does not fall in the air is that it is affected by the force and following the Bernoulli principle. On this basis, the conditions required for the propeller rotation are explored through propeller rotation experiments. Third, providing solutions and plans to the problems and needs. Using the concept of scale to draw propeller aircraft drawings in prescribed area. Fourth, selecting the optimal solution. The team members will negotiate and determine the final propeller aircraft design drawings for their group based on aircraft model materials. Fifth, building the aircraft model according to the design drawings and take field tests. The team members will build the aircraft model by cooperation according to the experimental precautions. After the model is completed, the test flight will be conducted under the guidance of the teachers. Sixth, estimating the design. To explore the flight test results, optimize the aircraft model, and complete the model flight competition. Seventh, improving the design. Team members conduct brainstorming to further optimize the aircraft design drawings. Eighth, sharing the design. Each team shared the concept, role and value of their team ' s aircraft design drawings.

## Developing a Curriculum Based on the Framework

Course manuals for teachers and students are provided. The teacher's manual presents a wealth of resources and guides which provide sets of flexible options for teaching. The students' handbook offers multiple question frames and worksheets which encourage the habit of recording and reflecting on experimental processes.

The course takes aircraft as the theme and addresses the core topic of constructing an airplane. Areas covered include the invention of airplanes, the principles of aircraft flight, aircraft design, assembling aircraft, flying aircraft, intelligent aircraft systems, and new progress in aerospace. The process by which students worked out practical problems to problems in engineering design drew on the modules presented in Table 1.

**Table 1:** Course content of *Soaring in the Air*.

**Modules**	**Class time**	**Disciplinary themes**	**Aims and content of modules**	**Related disciplines**
Module 1	2 h	The history of invention	Compare the speed of different vehicles and learn the formula “Velocity=Acceleration/Time” and its conversion formula.	Science Humanities Mathematics
			Understand aircraft types and emphasize the similarities and differences between propeller and jet aircraft.	
			Understand the history of the birth of airplanes; cultivate scientific thinking and the scientific spirit of persistence.	
			Learn the process of manufacturing aircraft and the work of aircraft manufacturing engineers; cultivate the spirit of scientific exploration.	
Module 2	2 h	The principles of aircraft	Learn the concept of force; understand the components and functions of the aircraft.	Science Mathematics
			Analyze the force of aircraft and distinguish between universal gravitation and gravity.	
			Grasp Bernoulli's principle and thoroughly understand its connotations by conducting small experiments.	
			Make a paper airplane that flies steadily and far; understand the force of the airplane and Bernoulli's principle.	
			Probe the factors affecting the flight distance of aircraft and improve scientific quality.	
Module 3	2 h	The design of aircraft	Identify and analyze tasks to stimulate interest in learning.	Mathematics Engineering
			Understand the spiral and jet power system and formulate the design plan.	
			Grasp the concept of measuring scale and determine the design plan according to the engineering design process.	
			Evaluate the design plan and develop a scientific and rigorous engineering attitude.	
Module 4	2 h	Assembling and test	Deepen the understanding of each part of the aircraft and its functions by assembling the aircraft.	Technology Engineering
			Discover the problems during flight test activities and find solutions.	
			Motivate the awareness of competition through model airplane contests; cultivate class unity and cooperation with peers.	
			Clarify the design plan and explain the existing problems of the aircraft; suggest solutions to these; develop skills in personal expression and cooperation in group activities.	
Module 5	2 h	Aircraft	Understand the meaning and layered structure of the atmosphere, distinguish between aircraft and spacecraft, and select aircraft suited to each layer of the atmosphere.	Science Humanities
			Design future aircraft according to the research steps of bionics.	
			Check mastery of the course content through the “you draw and I guess” game.	
Module 6	2 h	Aerospace	Learn about international and national achievements in aerospace and aviation.	Humanities
			Draw the theme of “Flying Dream”, cultivate imagination, stimulate aerospace dreams, and interest in aerospace exploration.	

## Methods

### Participants

This study was conducted as part of the “STEAM Plus” curriculum project carried out in the Huairou District of Beijing between December 28, 2020 to January 15, 2021. A total of 164 third-grade pupils were randomly selected to participate. Boys accounted for 52.4% (*n* = 86), while girls constituted 47.6% of the sample (*n* = 78). No participant had any previous experience of a course informed by the STEAM/Maker integrated curriculum. The research team spent 2 weeks teaching students on the self-developed STEAM and Maker integrated course *Soaring in the Air*.

Students study *Soaring in the Air* course at the Maker Lab. The desks of students in the Maker Lab are assembled and placed in groups. The Maker Lab is equipped with different kinds of experimental materials and tools that students need in the course learning, such as materials needed for Bernoulli principle proof experiments, aircraft model kits, etc.

### Instruments

The learning motivation scale used in this study was adapted from the ARCS motivation model proposed by Keller (2009). The model has demonstrably excellent levels of reliability and validity in evaluating students' learning motivation. The strong factor structure of the entire toolset allows for this reduction in the item count. So it contains a total of 33 items, 17 of which were used for the study, in accordance with the developmental ages of the participants. These included six items on the dimension of attention, four each on relevance and confidence, and three items on satisfaction. Responses are graded on a 5-level Likert scale, with “1” indicating complete disagreement and the remaining numbers signifying increasingly full agreement with each statement. Findings from a small pilot study confirmed the scale's strong reliability and validity (α = 0.891, *KMO* = 0.789).

Measurement of self-efficacy used an adapted version of the General Self-efficacy Scale (GSES) developed by Zhang and Schwarzer (1995). It consists of a total of 10 items with single-dimensional scales. Responses to each question are recorded on a 4-level Likert scale, from completely incorrect (1), to “somewhat,” “mostly,” and “completely” correct (2–4). The pre-experimental results demonstrated high levels of reliability and validity (α = 0.793; *KMO* = 0.709).

The STEAM test questions were adapted from a multi-disciplinary test bank. The question types and scores consisted of seven multiple-choice questions, each worth five points; four gap-fill questions containing eight blanks, with five points per blank; and one link question worth 25 points.

The process task list is independently developed by the research team according to the course content, which mainly includes five dimensions: S (Science), T (Technology), E (Engineering), A (Art), M (Mathematics). Each dimension is scored 5 points, 3 points, 1 point and 0 points. Completing all tasks as required were scored 5 points. Completing half of the tasks were scored three points. Completing <20% of the tasks were scored 1 point, and no answer was 0.

### Data Collection and Analysis

Two teachers were participated in teaching process. Teacher 1 was mainly responsible for completing the classroom teaching task according to the teaching design. Teacher 2, as an assistant, cooperates with the teacher 1 to complete the demonstration process of scientific inquiry experiment. Teacher 2 was mainly responsible for the distribution of experimental materials and task sheets, providing students guidance in the process of completing hands-on activities and keeping the activity in order. To ensure the students had enough thinking time and activity space, both two teachers provided well-structured learning environment and self-efficacy development situation for students to deal with problems and scientific questions.

For statistical analyses, SPSS Statistics 22.0 was used. The first module measured the level of students' self-efficacy. During modules 2–5, students' procedural task lists were collected. In the 6 module measured students ' learning motivation, self-efficacy and the STEAM test questions. The procedural task list completed by students in the classroom was collected and manually graded by the research team according to shared criteria. The students' overall STEAM scores derived from their results for the final test and procedural task, each of which contributed 50% to their total score.

To understand whether students' learning motivation, self-efficacy, and acquisition of interdisciplinary STEAM knowledge developed as a result of the course, descriptive statistics were applied to the data. A paired-sample *T*-test was run to determine the self-efficacy changes before and after the course. An independent-samples *T*-test was run to determine the existence of any gender-specific effects.

## Results

### Learning Motivation

Table 2 displays the results of the analysis of learning motivation, which consists of four parts: Attention\Relevance\Confidence\Satisfaction. The mean values for the dimensions of total score, attention, relevance, and satisfaction were all >3, the boys score slightly higher than girls, indicating the high level of students' learning motivation after the course had ended, and the boys were marginally more interested in such integrated courses, which also indicated that the courses' overall ability to adapt to the learning needs of boys and girls. However, the mean value of the confidence dimension (*M* = 2.8979, *SD* = 0.7783) was between 2.5 and 3, and girls score slightly higher than boys, indicating that students' self-confidence had reached the upper-middle level after the course, and girls' self-confidence was slightly stronger than boys. This slightly lower result may reflect the fact the uncertainty of students who had never previously encountered this type of course. In view of the broad sample for the sake of completeness, gender effects were also calculated. No gender impact appeared (*t*-test no sig). This result confirmed that the suitability of the *Soaring in the Air* course for motivating students in large-scale, gender-inclusive teaching environments.

**Table 2:** Learning motivation of students.

	**Dimensions**		***M***	***SD***	***n***	***t***
Learning motivation	Attention	Whole	3.3110	0.5677	164	–
		Boy	3.3353	0.58158	86	0.574
		Girl	3.2842	0.55447	78	
	Relevance	Whole	3.9741	0.9158	164	–
		Boy	4.0610	0.94163	86	1.279
		Girl	3.8782	0.88250	78	
	Confidence	Whole	2.8979	0.7783	164	–
		Boy	2.8547	0.83646	86	−0.746
		Girl	2.9455	0.72373	78	
	Satisfaction	Whole	4.2846	0.9292	164	–
		Boy	4.2907	0.97792	86	0.089
		Girl	4.2778	0.87850	78	
	Total score	Whole	3.5416	0.5666	164	–
		Boy	3.5616	0.62218	86	0.472
		Girl	3.5196	0.50142	78	

### Self-Efficacy

Table 3 presents the results of the analysis of self-efficacy. The post-test mean levels of students' self-efficacy (*M* = 3.179, *SD* = 0.5854) was higher than the pre-test score (*M* = 3.068, *SD* = 0.5475). A paired-sample *t*-test was performed on the pre- and post-test data, with the results showing that the difference between the two mean values was statistically significant (*p* = 0.015 < 0.05). In view of the broad sample for the sake of completeness, gender effects were also calculated. No gender impact appeared (*t*-test no sig), which was consistent with the findings on motivation. It is tentatively suggested that the boys felt marginally more able to adapt to the integrated syllabus than the girls in this study: more conclusively, the *Soaring in the Air* course appears well-adapted to the simultaneous teaching of boys and girls.

**Table 3:** Self-efficacy of students.

	**Dimensions**		***M***	***SD***	***n***	***t***
Self-efficacy	Pre-test		3.068	0.5475	164	−2.462^*^
		Whole	3.179	0.5854	164	
	Post-test	Boy	3.191	0.6110	86	
		Girl	3.167	0.5594	78	

* *p <0.05*.

### Analysis of STEAM Scores

Table 4 indicates students' acquisition of interdisciplinary knowledge following the course. The mean value of students' STEAM scores was 65.46 points, demonstrating that students had acquired an upper-middle level of interdisciplinary knowledge. For students new to interdisciplinary integrated curriculum learning, this was an impressive achievement. Girls scored slightly higher than boys, but again, there was no obvious discrepancy in performance. In fact, the primary conclusion to be drawn is that the interdisciplinary content and pedagogic approach of the *Soaring in the Air* course benefited both male and female participants in the study.

**Table 4:** Analysis of STEAM scores.

	**Dimensions**	***M***	***SD***	***n***	***t***
STEAM scores	Whole	65.46	14.921	164	–
	Boy	65.06	14.4	86	−0.359
	Girl	65.90	15.558	78	

## Discussion

### The Effects of Curriculum on Students' Learning Motivation and Self-Efficacy and Knowledge Acquisition

The study results demonstrated positive changes to students' learning motivation and self-efficacy. These findings resonate with previous studies showing that the students offered a genuinely creative learning environment demonstrate improvements in their attitudes to learning and their persistence (Kong and In-Cheol, 2014; Engelman et al., 2017). They also confirm that STEAM education based on school-oriented science textbooks can boost students' motivation (Bae et al., 2013; Choi, 2013; Bahri et al., 2017) and support the development of self-efficacy (Kong and Huo, 2014). The *Soaring in the Air* course connects interdisciplinary concepts with life experience to create a diversified learning environment where students can experience the joy of using their hands and brains while learning knowledge and skills. Burguillo (2010) points out that the type of positive competition encouraged throughout our course can support the motivation to learn. Moreover, the competitive relationship between groups also helps students to actively construct scientific knowledge, promote their subjectivity and initiative, and further elevate their motivation and self-efficacy.

The findings also indicate that students successfully acquired the interdisciplinary knowledge integrated into the framework of engineering design by the *Soaring in the Air* course. In solving practical problems, students developed their awareness of the relationship between different disciplinary viewpoints. This process generates higher-level understandings of science (Ivanitskaya et al., 2002), ultimately building students' interdisciplinary knowledge. These findings corroborate previous studies evaluating the effects of an integrated STEAM approach on learning. For instance, it has been found that STEAM pedagogies boost students' ability to conceptualize themes (Liliawati et al., 2018), improve the acquisition of concepts (Perignat and Katz-Buonincontro, 2019; Wandari et al., 2019; Ozkan and Topsakal, 2020), enhance disciplinary knowledge (Ceylan and Ozdilek, 2015), raise test scores (Chien and Chu, 2018) and benefits overall academic performance (Kim et al., 2014). The current study aligns with these results, finding that STEAM courses supported by Maker technology within the framework of engineering design can increase students' academic motivation and self-efficacy, thereby facilitating the acquisition of interdisciplinary knowledge.

### Curriculum Are Inclusive

The differences between boys and girls in this study were minor. Boys were marginally more motivated and achieved slightly higher scores in self-efficacy, with girls scoring fractionally higher on their STEAM scores. Nevertheless, the gender gap is manifested in the less positive attitudes and interests in STEM fields held by girls (Wang et al., 2019), and there are also discrepancies in the understanding of concepts between male and female students (Sagala et al., 2019). Women account for a relatively low proportion of roles in STEM professions (Beede et al., 2011; Weber, 2012; Su and Rounds, 2015; Casad et al., 2018; Rainey et al., 2018; García-Holgado et al., 2020). Thus, even the small differences recorded in this study should be taken into consideration as potential indicators that the STEM gender gap may begin early and widen with age.

Courses such as *Soaring in the Air* have prominent educational effects (Lee et al., 2013), which may reduce the academic and professional gender gap in STEM (Chachashvili-Bolotin et al., 2016). The *Soaring in the Air* syllabus stimulated the enthusiasm of male and female students alike, improving their self-efficacy, and promoting the acquisition of interdisciplinary knowledge. The course could allow female students to experience their skills and competences unbiasedly. The course content of *Soaring in the Air* is systematic, the course activities are universal, the course links are flexible, and the course itself is highly adaptable to the learning needs of every student. These findings resonate with those of MacPhee et al. (2013), who investigated the academic self-efficacy of STEM students. The authors discovered that the academic self-efficacy of female students was lower than that of male students upon enrollment in an interdisciplinary STEM course, but this difference had disappeared by the time they graduated.

## Conclusions

The goal of STEAM education is to strengthen learning in individual subjects (Blackley and Howell, 2015) to produce new understandings and achievements which transcend any single discipline (Peppler and Wohlwend, 2018). It also aims to improve students' creativity and ability to solve real-world problems (Watson and Watson, 2013; Kant et al., 2018). However, existing approaches to STEAM are often little more than an agglomeration of school subjects. Contemporary brain science has confirmed the importance of using hands in the learning process (Dougherty, 2012), which aligns with the idea of “learning by making” central to Maker education. This approach prizes creativity and innovation, but its prioritization of technology over principles is a major hindrance to cultivating such qualities in students.

This research designed an integrated STEAM and Maker approach to primary education by utilizing the framework of engineering design. The students' academic motivation, self-efficacy, and acquisition of cross-disciplinary knowledge were measured at high levels after the course. Moreover, the fact that no obvious difference between male and female students was identified testifies to the gender inclusivity of *Soaring in the Air*.

Based on the results, we recommend that further courses integrating STEAM and Maker approaches be developed using the expertise of researchers and curriculum developers. We furthermore propose that STEAM teachers focus on teaching goals that are comprehensible to students and can access a toolkit of teaching methods appropriate to the course content. Students should be confronted with real-world problems and situations which encourage them to connect their learning with the empirical world beyond the classroom. As Brooks and Brooks (1993) pointed out, it is only when learners associate prior knowledge with new experience and new skills in a real environment that meaningful learning will occur. It is also necessary to consider how to integrate Chinese, mathematics, physics, chemistry and other classes into STEAM courses, and how to cultivate students' passion for science. We believe that if resources are allocated to developing inclusive STEAM courses and the expertise of teachers in the future, the quality of STEAM education will continue to improve.

## Data Availability Statement

The original contributions presented in the study are included in the article/Supplementary Files, further inquiries can be directed to the corresponding author/s.

## Ethics Statement

Approval by an ethics committee was not required for this study as per applicable institutional and national guidelines and regulations. All participating students expressed their willingness to participate in this activity.

## Author Contributions

All authors listed have made a substantial, direct and intellectual contribution to the work, and approved it for publication.

## Funding

This work was supported by 2019 Ministry of Education Humanities and Social Sciences Research Planning Fund Project' Research on the Integration Path of STEAM and Maker in Primary School Science Education' (Project No. 19YJA880091).

## Conflict of Interest

The authors declare that the research was conducted in the absence of any commercial or financial relationships that could be construed as a potential conflict of interest.

## Publisher's Note

All claims expressed in this article are solely those of the authors and do not necessarily represent those of their affiliated organizations, or those of the publisher, the editors and the reviewers. Any product that may be evaluated in this article, or claim that may be made by its manufacturer, is not guaranteed or endorsed by the publisher.
