# Supplementary material for: A Curriculum Integrating STEAM and Maker Education Promotes Pupils' Learning Motivation, Self-Efficacy, and Interdisciplinary Knowledge Acquisition
Source: Front Psychol. 2021 Sep 8;12:725525. doi: 10.3389/fpsyg.2021.725525 (PMC8455939; doi:10.3389/fpsyg.2021.725525)
Supplement: Supplementary file 1 [file Table_1.docx]

# APPENDIX

# TABLE 1| Course content of *Soaring in the Air*

| Modules | Class time | Disciplinary themes | Aims and content of modules | Related disciplines |
| --- | --- | --- | --- | --- |
| Module 1 | 2 hours | The history of invention | Compare the speed of different vehicles and learn the formula "Velocity=Acceleration/Time" and its conversion formula. | Science  Humanities  Mathematics |
|  |  |  | Understand aircraft types and emphasize the similarities and differences between propeller and jet aircraft. |  |
|  |  |  | Understand the history of the birth of airplanes; cultivate scientific thinking and the scientific spirit of persistence. |  |
|  |  |  | Learn the process of manufacturing aircraft and the work of aircraft manufacturing engineers; cultivate the spirit of scientific exploration. |  |
| Module  2 | 2 hours | The principles of aircraft | Learn the concept of force; understand the components and functions of the aircraft. | Science  Mathematics |
|  |  |  | Analyze the force of aircraft and distinguish between universal gravitation and gravity. |  |
|  |  |  | Grasp Bernoulli's principle and thoroughly understand its connotations by conducting small experiments. |  |
|  |  |  | Make a paper airplane that flies steadily and far; understand the force of the airplane and Bernoulli's principle. |  |
|  |  |  | Probe the factors affecting the flight distance of aircraft and improve scientific quality. |  |
| Module 3 | 2 hours | The design of aircraft | Identify and analyze tasks to stimulate interest in learning. | Mathematics  Engineering |
|  |  |  | Understand the spiral and jet power system and formulate the design plan. |  |
|  |  |  | Grasp the concept of measuring scale and determine the design plan according to the engineering design process. |  |
|  |  |  | Evaluate the design plan and develop a scientific and rigorous engineering attitude. |  |
| Module 4 | 2 hours | Assembling and test | Deepen the understanding of each part of the aircraft and its functions by assembling the aircraft. | Technology  Engineering |
|  |  |  | Discover the problems during flight test activities and find solutions. |  |
|  |  |  | Motivate the awareness of competition through model airplane contests; cultivate class unity and cooperation with peers. |  |
|  |  |  | Clarify the design plan and explain the existing problems of the aircraft; suggest solutions to these; develop skills in personal expression and cooperation in group activities. |  |
| Module 5 | 2 hours | Aircraft | Understand the meaning and layered structure of the atmosphere, distinguish between aircraft and spacecraft, and select aircraft suited to each layer of the atmosphere. | Science  Humanities |
|  |  |  | Design future aircraft according to the research steps of bionics. |  |
|  |  |  | Check mastery of the course content through the "you draw and I guess" game. |  |
| Module 6 | 2 hours | Aerospace | Learn about international and national achievements in aerospace and aviation. | Humanities |
|  |  |  | Draw the theme of "Flying Dream", cultivate imagination, stimulate aerospace dreams, and interest in aerospace exploration. |  |
